# Acute disseminated encephalomyelitis mimicking late CNS relapse of acute lymphoblastic leukaemia: case report

**DOI:** 10.1186/1752-1947-1-4

**Published:** 2007-02-09

**Authors:** Ram Kumar, Shobha Nijalingappa, John Grainger, Omar Ismayl

**Affiliations:** 1 Department of Paediatric Neurology, Royal Manchester Children's Hospital, Hospital Road, Manchester, UK; 2 Department of Paediatric Haematology and Oncology, Royal Manchester Children's Hospital, Hospital Road, Manchester, UK

## Abstract

**Background:**

Acute encephalomyelopathy occurring after an allogeneic bone marrow transplant for leukaemia is a diagnostic emergency. The diagnosis can be challenging since there is a wide set of alternative diagnoses, including opportunistic infections and relapse of the leukaemia.

**Case presentation:**

A 13-year old girl presented with a severe acute myelopathy and encephalopathy. She was in prolonged remission from a central nervous system and bone marrow relapse of an acute lymphoblastic leukaemia, treated with allogeneic bone marrow transplantation. Neuroimaging showed multifocal grey and white matter lesions of demyelinating appearance in the brain and entire spine. Immunophenotyping and cytogenetic investigations of the girl's cerebrospinal fluid lymphocytosis excluded a late central nervous system relapse of her leukaemia. The diagnosis was acute disseminated encephalomyelitis. With standard immunosuppressive therapy, the girl had early cerebral recovery but a prolonged period of recovery from her myelopathy.

**Conclusion:**

Acute disseminated encephalomyelitis should be considered in the differential diagnosis of acute encephalomyelopathy after bone marrow transplantation for leukaemia. Demyelinating syndromes such as acute disseminated encephalomyelitis may be late sequelae of bone marrow transplantation.

## Background

Acute encephalomyelopathy occurring after bone marrow transplantation for leukaemia is a diagnostic emergency. The diagnosis is challenging since the differential is wide, including opportunistic infections and leukaemia recurrence [1]. Acute disseminated encephalomyelitis (ADEM) is an uncommon idiopathic immune-mediated demyelinating disorder, recognised as a cause of encephalomyelopathy in previously well children [2]. We report a child with acute disseminated encephalomyelitis occurring late after successful allogeneic bone marrow transplantation for an acute leukaemia whose presentation mimicked a previous CNS leukaemic relapse.

## Case presentation

A 13-year old girl presented with a rapidly progressive paralysis and encephalopathy. She had a mild viral-like illness for the preceding week, with lethargy. Over the days preceding presentation she developed back pain and difficulty in walking. On the morning of admission, she developed a headache, vomiting, a fluctuating level of consciousness and became unable to move her limbs.

Neurological examination showed an encephalopathic girl, with four limb paralysis, absent deep tendon and abdominal reflexes, and mild bilateral facial weakness. A high thoracic (C4) sensory level, severe urinary retention and stool incontinence was evident. Systemic examination did not reveal pyrexia, rash, lymphadenopathy, hepatosplenomegaly or sepsis.

Her remote clinical history was notable: at age 2 years, she had developed acute lymphoblastic leukaemia (ALL), common B-cell variant. Six months after completing chemotherapy using the UKALLXI protocol, the girl re-presented with an encephalopathy due to a CNS and bone marrow relapse of the leukaemia. The girl received cranial irradiation and further chemotherapy on the MRC UKALL R2 relapse protocol which achieved a further remission. Because of the high risk of further relapses, she proceeded to have an bone marrow transplant (BMT) with a matched unrelated male donor. Total body irradiation was used in conditioning for the BMT. Following the BMT, the girl was in prolonged remission for the following 7 years with no overt CNS or systemic sequelae.

**Figure 1:**
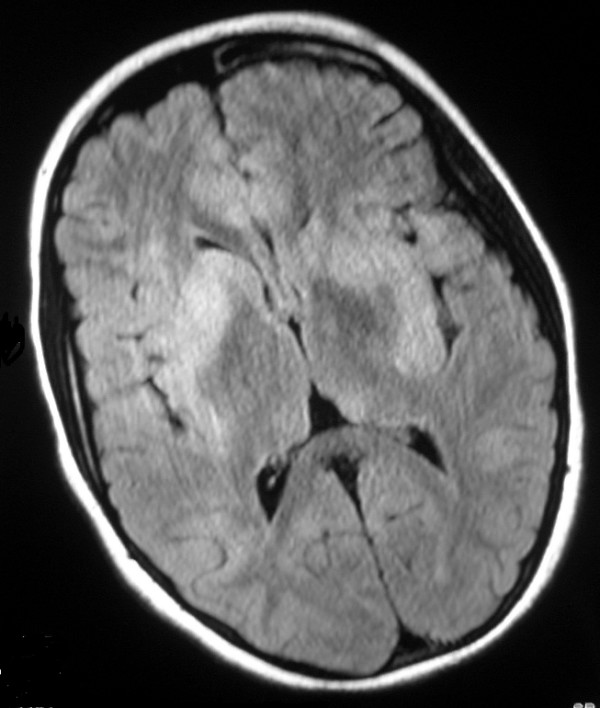
**Axial FLAIR sequence magnetic resonance image of brain at admission**. There are hyperintense multifocal lesions in the deep grey nuclei, subcortical white matter and cortex.

**Figure 2:**
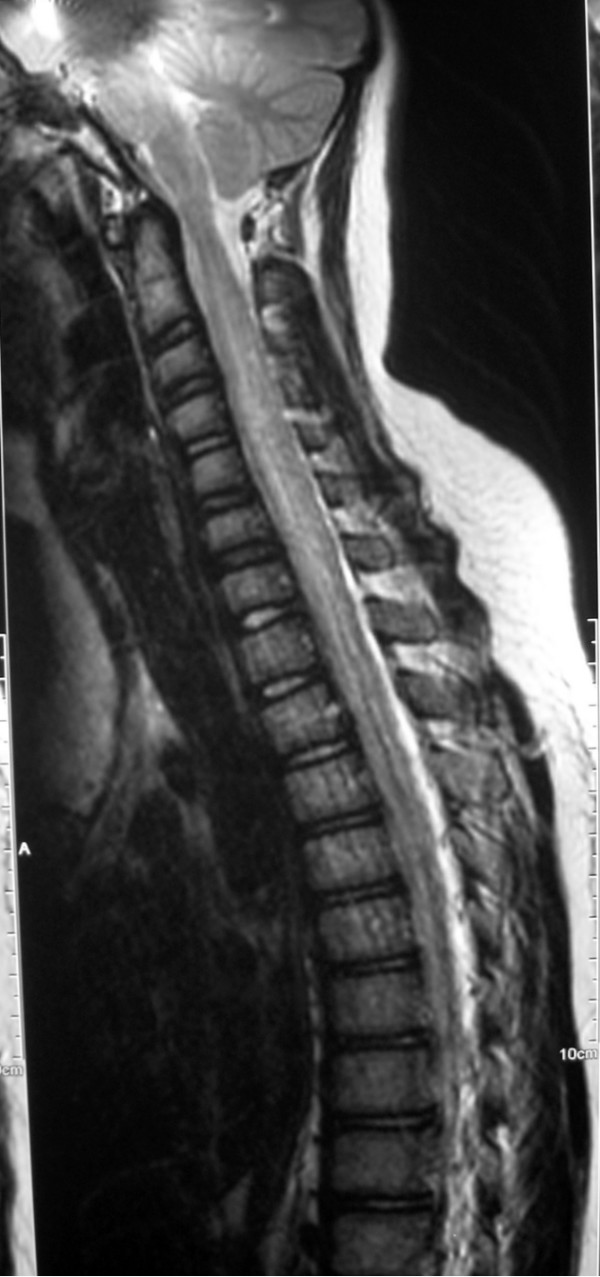
**Sagittal T2-weighted magnetic resonance image of spine at admission**. There is longitudinal hyperintense signal involving the central cord from C1 downwards.

Initial MR imaging showed diffuse involvement of the CNS (Figures 1 and 2). The spinal cord was diffusely swollen showing central cord T2 hyperintensity from C1 to the conus. There were multifocal grey and white matter lesions in the cerebral cortex, subcortical areas and cerebellum which showed diffuse gadolinium enhancement. The appearances were consistent with an infective or inflammatory encephalomyelitis, but the girl's remote history and presentation raised the concern of a recurrent CNS relapse of ALL.

**Figure 3:**
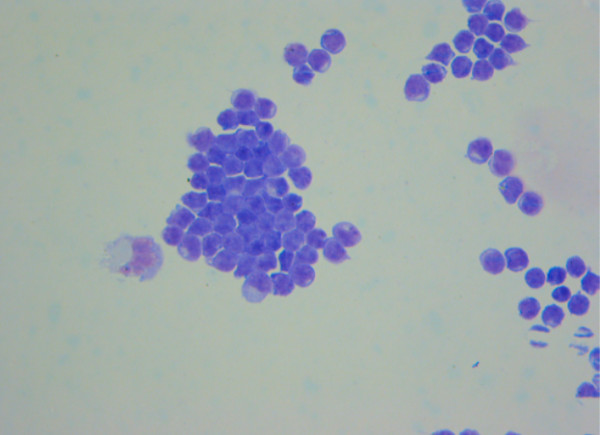
**CSF cytospin (x50 magnification)**. CSF cytospin with Giemsa's stain showing pronounced lymphocytosis.

CSF examination revealed 125 white cells with a lymphoblastic appearance and 5 red cells. CSF protein (0.61 g/l) and CSF:blood lactate ratio (3.2:1.2 mmol/l) were raised with a low CSF: glucose ratio (4.3:8.9 mmol/l). CSF cytospin showed increased proportion of lymphocytes which further increased suspicion of ALL relapse (Figure 3). Immunophenotyping of the CSF cells demonstrated the cells were CD10 negative, strongly CD2 and CD7 positive and terminal deoxynucleotidyl transferase (TdT) negative. These findings suggested that the cells were mature T-cells. Cytogenetic studies and FISH using centromeric X and Y-chromosome markers on the CSF lymphocytes confirmed that the overwhelming majority of cells were of male donor type (6–14% of cells were of host origin but not clonal). These CSF findings, along with a normal peripheral blood count and normal bone marrow biopsy confirmed that the girl's illness was not due relapse of her previous ALL.

The girl was treated with intravenous high-dose methylprednisolone and aciclovir. Blood-brain barrier studies showed an abnormal CSF IgG index; oligoclonal bands were not detected. Further negative tests included: CSF culture; CSF PCR for HSV1 and 2, VZV, EBV, HHV6/7, adenovirus, echovirus, parechovirus, enterovirus and echovirus; serology for influenza, mycoplasma, Chlamydia, and toxoplasma; ASOT, ANA, ANCA, anticardiolipin antibodies, tissue autoantibodies. Immune function tests did not reveal an underlying inmmunosuppression. A presumptive diagnosis of ADEM with associated transverse myelitis was made.

The girl's encephalopathy resolved over succeeding days without overt cerebral sequelae, but spinal recovery was much slower. A repeat MRI at 2 weeks after onset showed resolving brain and spinal lesions. Her disability slowly improved over several months: Barthel activities of daily living index was 15/100 at 3 weeks, 40/100 at 7 weeks, and 85/100 at discharge from hospital 3 months after onset. At last review, 10 months after onset, her Barthel index was 100/100. She was mobile on her feet without aids, and had just ceased intermittent catheterisation for urine voiding. She was unable to walk on her heels, and had lingering fatiguability.

## Discussion

This girl presented with the features of a severe acute encephalomyelopathy. We made the diagnosis of ADEM based on her MR imaging and exclusion of competing diagnoses. Being a diagnosis of exclusion, a discussion of ADEM entails discussion of the differential diagnosis (Table 1).

**Table 1:** Differential diagnosis of acute encephalomyelopathy after bone marrow transplantation for leukaemia

**Metabolic, nutrient and electrolyte disturbances**
**Treatment side-effects**
Cyclosporin (posterior leukoencephalopathy syndrome)
Amphotericin (parkinsonism)
Radiation sequelae (arteriopathy, vacuolating encephalomyelopathy)
**Infections**
Viruses (HSV, VZV, CMV, EBV, HHV6, HHV7, JC, BK, adenovirus, West Nile Virus)
Parasites (Toxoplasma, amoeba)
Fungi (Aspergillus, Candida)
Bacteria (abcesses, Listeria, Mycoplasma, TB)
**CNS relapse of leukaemia**
**Inflammation**
Acute disseminated encephalomyelitis
Multiple sclerosis
Vasculitides (SLE, CNS angiitis)
**Haemorrhage/infarction**
Thrombocytopoenic thrombotic purpura
Secondary to radiation arteriopathy
Idiopathic subarachnoid and subdural haemorrhage

An isolated CNS relapse of acute leukaemia was the initial concern because of the girl's previous CNS relapse. Concern was heightened by her marked CSF lymphocytosis. Isolated CNS relapse of leukaemia is uncommon after BMT in prolonged remission [3]. The MRI appearance that would be expected in CNS relapse of leukaemia is meningitic contrast enhancement due to leukaemic infiltrates, rather than the non-space occupying parenchymal lesions of the brain and intramedullary spine seen in this girl [4]. We demonstrated that the girl did not have a CNS relapse of her original common variant ALL using immunophenotyping and cytogenetics. Immunophenotyping, using fluorescent monoclonal antibodies, showed that the CSF cells lacked the CD10 antigen – a marker of immature common lineage lymphocytes which would be expected to be positive in a relapse. The cells demonstrated the CD2 and CD7 antigens which are both markers of T-cells. The absence of TdT, a marker of immature lymphocytes, showed the T-cells were mature reactive cells rather than lymphoblasts. The cytogenetic testing showed that the cells were not clonal, whereas clonal expansion would be expected in a relapse. Cytogenetic testing also showed the cells were derived mainly from the girl's male BMT donor, whereas in a relapse the cells should derive solely from the girl. The donor-host chimerism of these CSF T-cells is of note, since chimerism has been highlighted as a risk factor for CNS relapse after allogeneic BMT for leukaemia.

Other CNS sequelae of acute leukaemia and its treatment can also present with an acute encephalomyelopathy. These include: medication toxicity (e.g. cyclosporin), opportunistic CNS infection, secondary tumour, radiation myelopathy, mineralising arteriopathy, necrotising leukoencephalopathy and graft-versus-host-disease (GVHD) associated cerebral angiitis [1,4-6]. The majority of these CNS sequelae have been reported within the first 12 months after treatment, although secondary tumours typically appear later. The initial MRI appearance in this girl did not show the necrotizing or vacuolating appearance found in the radiation-related sequelae. GVHD-associated cerebral angiitis is an under-recognised entity which can appear as a late syndrome after allogeneic BMT. It can present with haemorrhagic or infarctive stroke, or a demyelinating encephalomyelitis with similar neuroimaging and CSF findings to those in our girl [5]. Unlike the previously reported patients with GVHD-associated cerebral angiitis, our girl did not have systemic features to suggest chronic GVHD.

Severe encephalomyelitis can be caused by herpesviruses, aspergillus and toxoplasmosis [6]. These infections are early rather than late sequelae, occurring during the period of immunosuppression. Myelitis due to these agents is uncommon in immunocompetent patients. It is not possible to distinguish infective encephalomyelitis from ADEM on MR imaging alone so we continued aciclovir treatment in our patient pending the results of virology investigations.

The combination of MR imaging appearances and exclusion of other diagnoses led to the diagnosis of ADEM [2]. The classical lesions of ADEM on MRI are multifocal lesions in the brain white matter, cortical grey matter and basal ganglia as in our girl. Around 20% of children with ADEM have spinal involvement, although total spinal cord length involvement as seen in our girl is atypical.

The immunophenotyping of the girl's prominent CSF lymphocytosis suggests that her ADEM was a T-cell driven disease process. This a novel finding, and is in keeping with a previous report of increased myelin reactivity in peripheral blood T-cells from children recovering from ADEM [7]. We suggest immunophenotyping with flow cytometry of CSF cells from patients with ADEM may reveal further insights into the immunopathogenesis of this condition.

There have been a few previous reports of children and adults with ADEM following BMT for various leukaemias and lymphomas [1,8,9]. All of these cases of ADEM occurred as an early sequel of allogeneic BMT, in the weeks or months of immunosuppression following the transplant. ADEM occurring several years after BMT, during prolonged remission, has not previously been reported.

We do not think it likely that the girl's ADEM was related to the combination of cranial and total body irradiation she received prior to her BMT. This combination has been associated with brain atrophy on neuroimaging and vascular sequelae (necrotizing leukoencephalopathy and cavernomatous angiodyslasias), but neurological sequelae overall were not significantly more common than in patients who did not receive cranial irradiation [1]. In addition ADEM and cerebral angiitis, both immunological sequelae, following BMT have been reported in patients who received only total body irradiation without cranial irradiation [5,9].

Could this girl have been predisposed to developing ADEM by her bone marrow transplantation? This is a feasible hypothesis. Autoimmune disorders may have an increased incidence after allogeneic BMT [10]. Guillain-Barré disease, a peripheral neurological disorder with an autoimmune component, has been reported as an early sequel in post-BMT patients [6]. As noted above, ADEM also has an autoimmune component with peripheral T-cells from children with ADEM show increased reactivity to host myelin basic protein [7]. It is possible that the BMT may induce a susceptibility to an environmental trigger for ADEM. We suggest that children who have received allogeneic BMT should be monitored for immune-mediated neurological disorders as part of long-term follow-up.

## Conclusion

Acute disseminated encephalomyelitis should be considered as an alternative to leukaemic relapse in patients with an acute encephalomyelopathy after allogeneic bone marrow transplantation. Demyelinating syndromes such as acute disseminated encephalomyelitis may be a late sequelae of bone marrow transplantation.

## Competing interests

The author(s) declare that they have no competing interests.

## Authors' contributions

RK and SN summarised the patient notes. RK and JG reviewed the existing literature. RK wrote the manuscript with review by JG and OI. All authors were involved in the clinical care of the patient. All authors read and approved the final manuscript.
